# Supplementary material for: Seismic evidence for a cold serpentinized mantle wedge beneath Mount St Helens
Source: Nat Commun. 2016 Nov 1;7:13242. doi: 10.1038/ncomms13242 (PMC5097125; doi:10.1038/ncomms13242)
Supplement: Supplementary Information — Supplementary Figures 1-3 and Supplementary References. [file ncomms13242-s1.pdf]

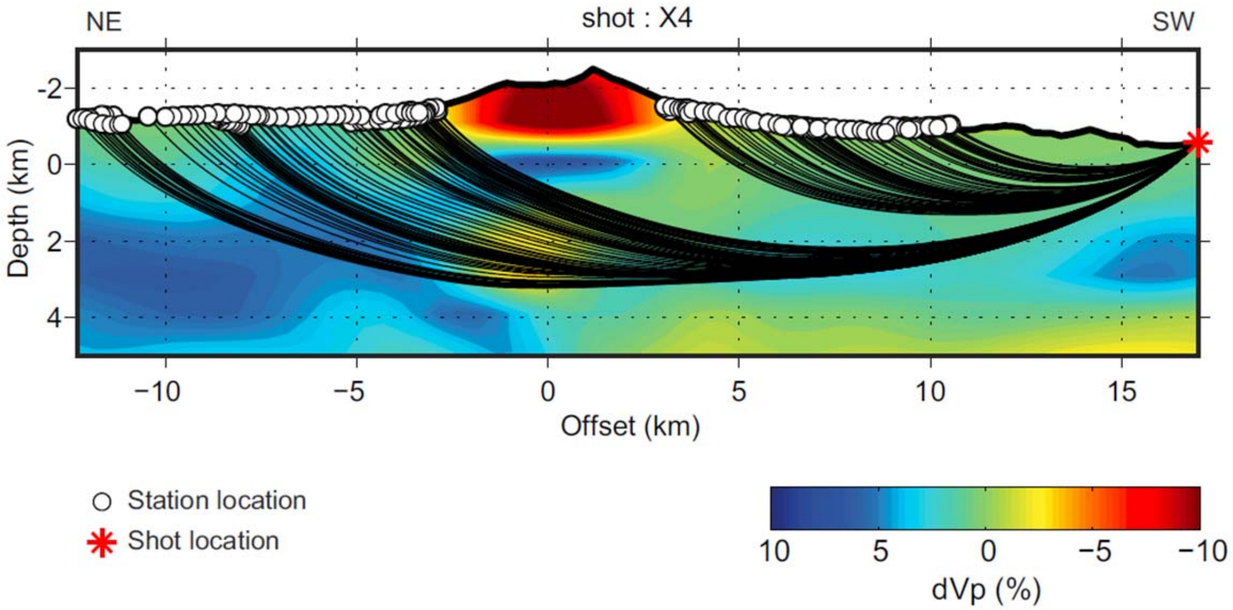

Supplementary Figure 1. An example travel time calculation for shot X4. The ray paths produced by the Fast Marching code[1,2] are drawn as black lines. The 3D tomography model used in the calculations[3] is plotted in the background as a perturbation about the 1D reference velocity model[4]. To calculate the full PmP travel time fields, the shallow 3D velocity model is embedded in the reference model and the Fast Marching algorithm is run to 60 km depth (not shown).

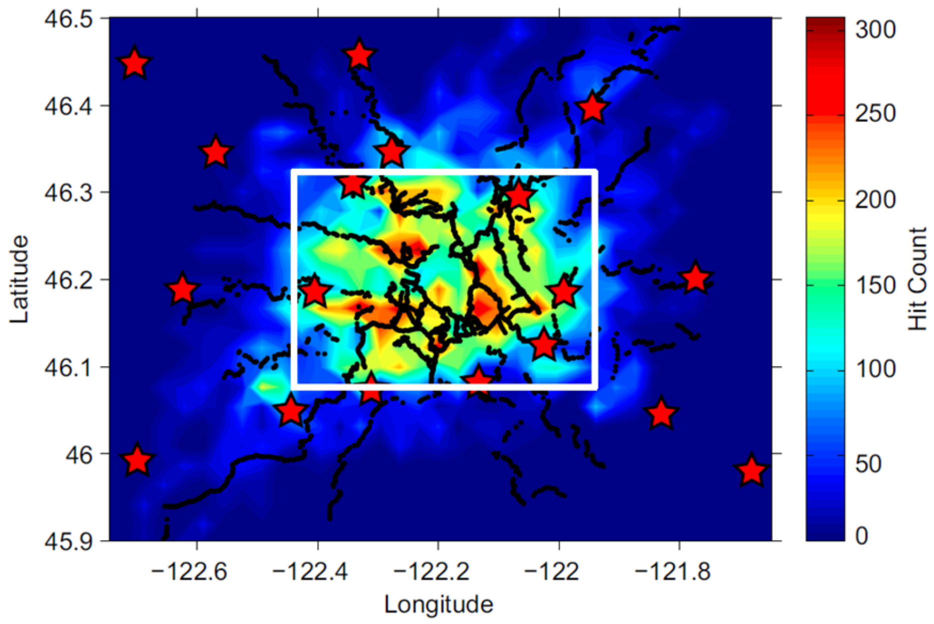

Supplementary Figure 2. A 2D hit count of source-receiver midpoint locations at Moho depth (40 km). The same binning parameters as the common midpoint (CMP) imaging are used. The white box denotes the location of the CMP image volume used in Figure 5 and was chosen to coincide with the region of greatest data fold. Station locations are plotted as black dots and shot locations are red stars.

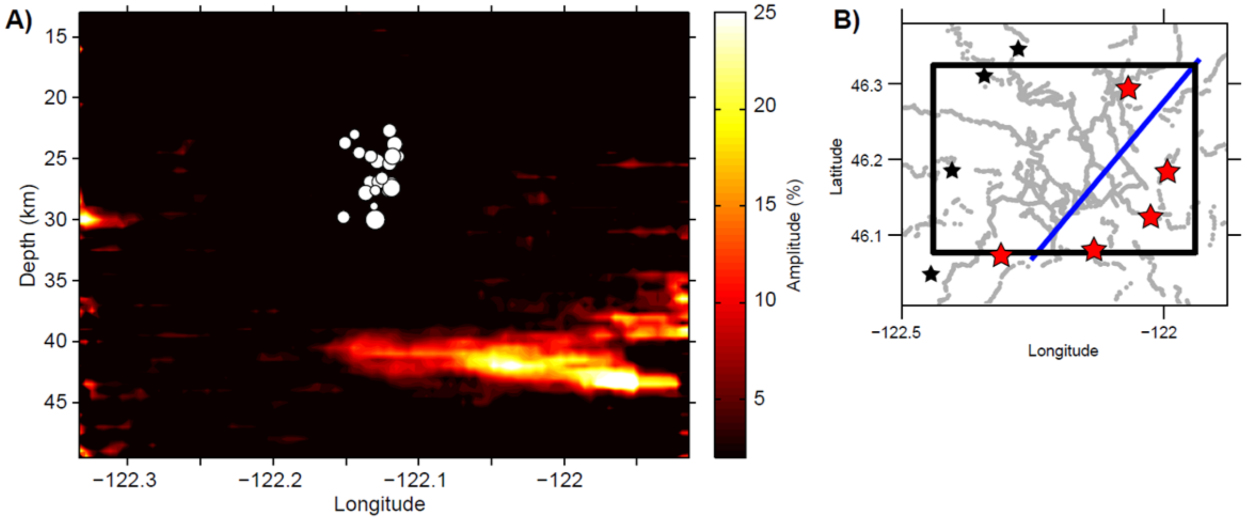

Supplementary Figure 3. CMP cross-section with deep long period earthquake locations. The location of the northeast trending cross section is shown in (**B**) as a blue line, the Moho imaging volume in Figure 5 is outlined in black, stations are plotted in gray and shots are stars. The image in (**A**) results from two dimensional common midpoint stacking using only the shots plotted in red and stations within 3 km of the cross section. White dots show nearby DLP earthquake locations projected onto the cross section and their size scales with event magnitude.

Supplementary References

1) Rawlinson, N., & Sambridge, M. Wave front evolution in strongly heterogeneous layered media using the fast marching method. *Geophys. J. Int.* **156**(3), 631–647 (2004).

2) Rawlinson, N., & Sambridge, M. Multiple reflection and transmission phases in complex layered media using a multistage fast marching method. *Geophysics* **69**(5), 1338–1350 (2004).

3) Waite, G. P., & Moran, S. C. VP Structure of Mount St. Helens, Washington, USA, imaged with local earthquake tomography. *J. Volcanol. Geotherm. Res.* **182**(1), 113–122 (2009).

4) Thelen, W. A., Crosson, R. S., & Creager, K. C. Absolute and relative locations of earthquakes at Mount St. Helens, Washington, using continuous data: implications for magmatic processes. *US Geological Survey professional paper* **1750**, 71-95 (2008).
